# Minimally Invasive Management of a Giant Pancreatic Pseudocyst Following Acute Pancreatitis: A Case Report and Literature Review

**DOI:** 10.7759/cureus.94564

**Published:** 2025-10-14

**Authors:** Roberto Elías Damacio-Bretón, Juan Manuel Horacio Montes-Salas, Marco Aurelio Alvarez-Romero, Brenda Sanchez-Melo, Alejandro Tenorio-Pacheco

**Affiliations:** 1 Department of General Surgery, Hospital de Especialidades 5 de Mayo, Instituto de Seguridad y Servicios Sociales de los Trabajadores al Servicio de los Poderes del Estado (ISSSTEP), Puebla, MEX; 2 Department of Medicine, Benemérita Universidad Autónoma de Puebla, Puebla, MEX; 3 Department of Surgery, Hospital de Especialidades 5 de Mayo, Instituto de Seguridad y Servicios Sociales de los Trabajadores al Servicio de los Poderes del Estado (ISSSTEP), Puebla, MEX; 4 Department of General Medicine, Hospital de Especialidades 5 de Mayo, Instituto de Seguridad y Servicios Sociales de los Trabajadores al Servicio de los Poderes del Estado (ISSSTEP), Puebla, MEX

**Keywords:** acute pancreatitis (ap), extrapancreatic pseudocyst, laparoscopic cystogastrostomy, laparoscopic extragastric cystogastrostomy, minimally invasive pancreatic surgery

## Abstract

Pancreatic fluid collections (PFCs) are a known complication of pancreatitis. According to the Revised Atlanta Classification, these can mature into either a pancreatic pseudocyst or walled-off necrosis (WON), the latter containing solid debris. While many collections resolve spontaneously, large, symptomatic, or complicated ones require intervention. Laparoscopic drainage is a key minimally invasive option. We present the case of a 23-year-old male patient with presumed alcohol-related pancreatitis who developed a giant 18.5 cm PFC. After six weeks of conservative management, a laparoscopic extragastric cystogastrostomy was performed due to persistent symptoms, draining 600 mL of necrotic material, suggesting the collection was consistent with WON. The patient was readmitted 20 days postoperatively for a peripancreatic abscess, which was managed medically. This case highlights the successful laparoscopic management of a large, complex PFC and underscores the importance of structured postoperative surveillance for infectious complications.

## Introduction

According to the Revised Atlanta Classification, pancreatic fluid collections (PFCs) are a common sequela of acute pancreatitis [1]. These collections can mature into two main types: a pancreatic pseudocyst (PPC), which is a homogeneous fluid collection encapsulated by a fibrous wall, or walled-off necrosis (WON), which contains a variable amount of solid necrotic debris [1,2]. This distinction is critical as it impacts management, though it can be challenging to differentiate preoperatively. PPCs and WON are frequent complications of pancreatitis, trauma, or ductal obstruction [3-7].

While many PFCs resolve spontaneously, intervention becomes necessary for collections that persist, increase in size, or become symptomatic [5,8]. Indications for drainage include pain, nausea, vomiting, or complications like infection or obstruction [3,8]. A size greater than 6 cm is a relative indication for intervention [6-8]. The current management algorithm has evolved toward minimally invasive techniques. Endoscopic ultrasound (EUS)-guided drainage is often first-line, while laparoscopic drainage is a primary alternative when EUS is unavailable, anatomically unfavorable, or has failed [5,8]. The rationale for this case report is to describe the management of a giant, complex PFC in a young adult where institutional limitations guided the therapeutic choice, highlighting a real-world application of the surgical step-up approach.

## Case presentation

A 23-year-old male patient presented on May 26, 2024, with severe, knife-like epigastric abdominal pain (10/10 Visual Analog Scale) and intractable vomiting. The etiology was presumed to be alcohol-related, given the patient's history; a comprehensive workup to formally exclude other causes, such as gallstones, hypertriglyceridemia, or autoimmune pancreatitis, was not detailed in the available admission records. At presentation, the patient was hemodynamically stable, with no signs of organ failure per the Marshall scoring system.

Upon admission, laboratory studies showed a marked systemic inflammatory response (Table 1). The patient was managed conservatively with intravenous fluid resuscitation (specific type and rate not recorded), analgesia, fasting, and nasogastric tube placement for decompression. Prophylactic antibiotics were not administered, and ICU admission was not required.

**Table 1:** Laboratory results on admission (June 3, 2024) ^*^Values outside the normal reference range

Parameter	Result	Reference value
Complete blood count		
Hemoglobin	11.8 g/dL	13.5-17.5 g/dL
Leukocytes	23.8 × 10³/µL^*^	4.5-11.0 × 10³/µL
Neutrophils	73.7%	40-75%
Platelets	244 × 10³/µL	150-450 × 10³/µL
Blood chemistry		
Glucose	116 mg/dL^*^	70-100 mg/dL
Creatinine	0.7 mg/dL	0.6-1.2 mg/dL
Enzymes and reactants		
Amylase	114 U/L^*^	28-100 U/L
C-reactive protein	318 mg/L^*^	<10 mg/L
Total bilirubin	2.37 mg/dL^*^	0.2-1.2 mg/dL

**Figure 1:**
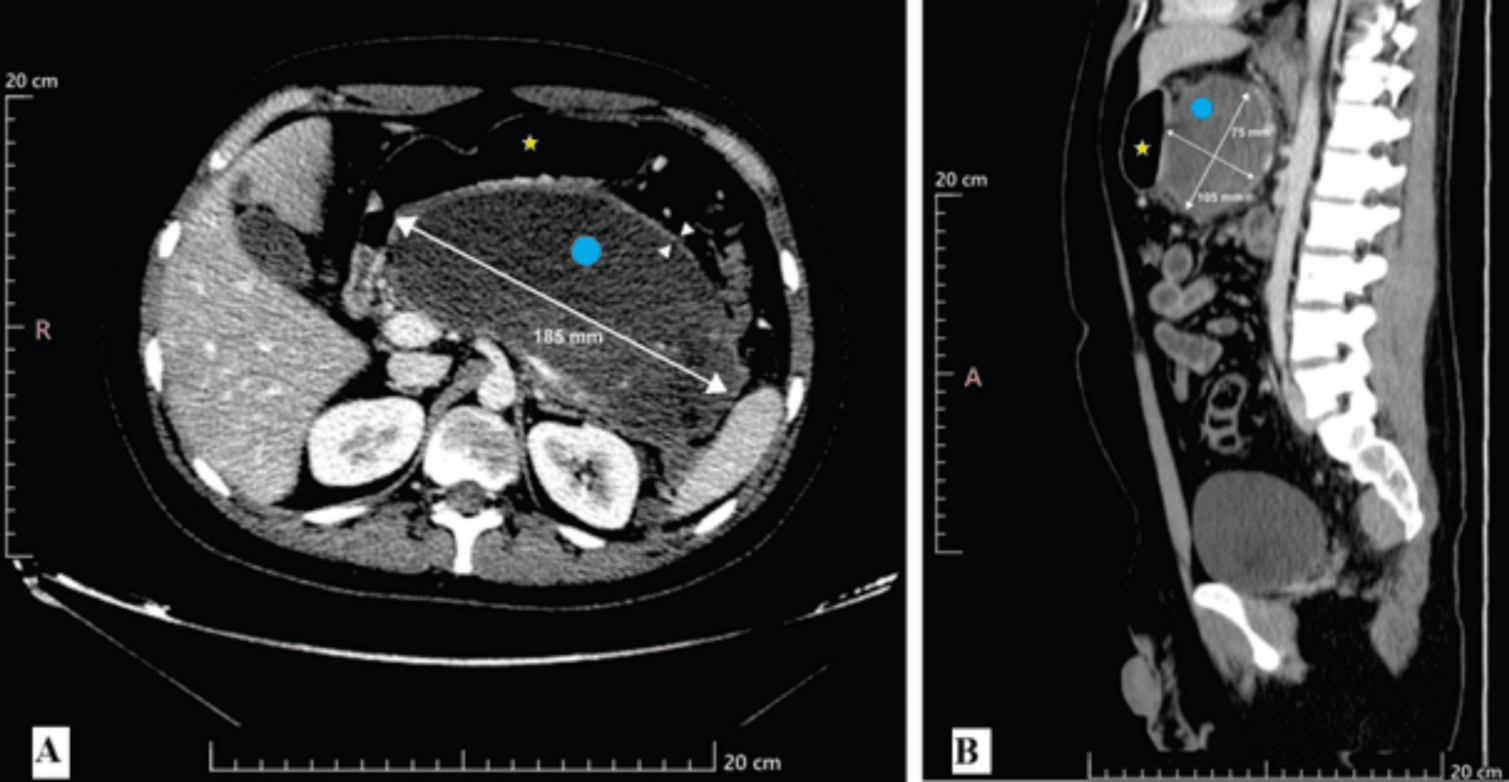
Contrast-enhanced computed tomography of the abdomen The axial view (A) and sagittal view (B) show a large pancreatic pseudocyst (cyan dot) posterior to the stomach (yellow star). The pseudocyst's dimensions are indicated by the measurement lines: 185 mm in the transverse plane, 75 mm in the anteroposterior plane, and 105 mm in the craniocaudal plane

A contrast-enhanced CT scan on June 3, 2024, revealed an enlarged pancreas and a large, hypodense, well-walled peripancreatic fluid collection measuring 185 x 74 x 105 mm (Figure 1). The CT did not show definitive nonenhancing solid components, and the lesion was initially characterized as a pseudocyst.

After six weeks, the collection had radiologically matured, but the patient's symptoms persisted. A surgical approach was decided upon as EUS-guided drainage was not available at our institution. A laparoscopic approach was deemed appropriate. The patient was classified as American Society of Anesthesiologists III due to the systemic inflammation and large fluid collection.

**Figure 2:**
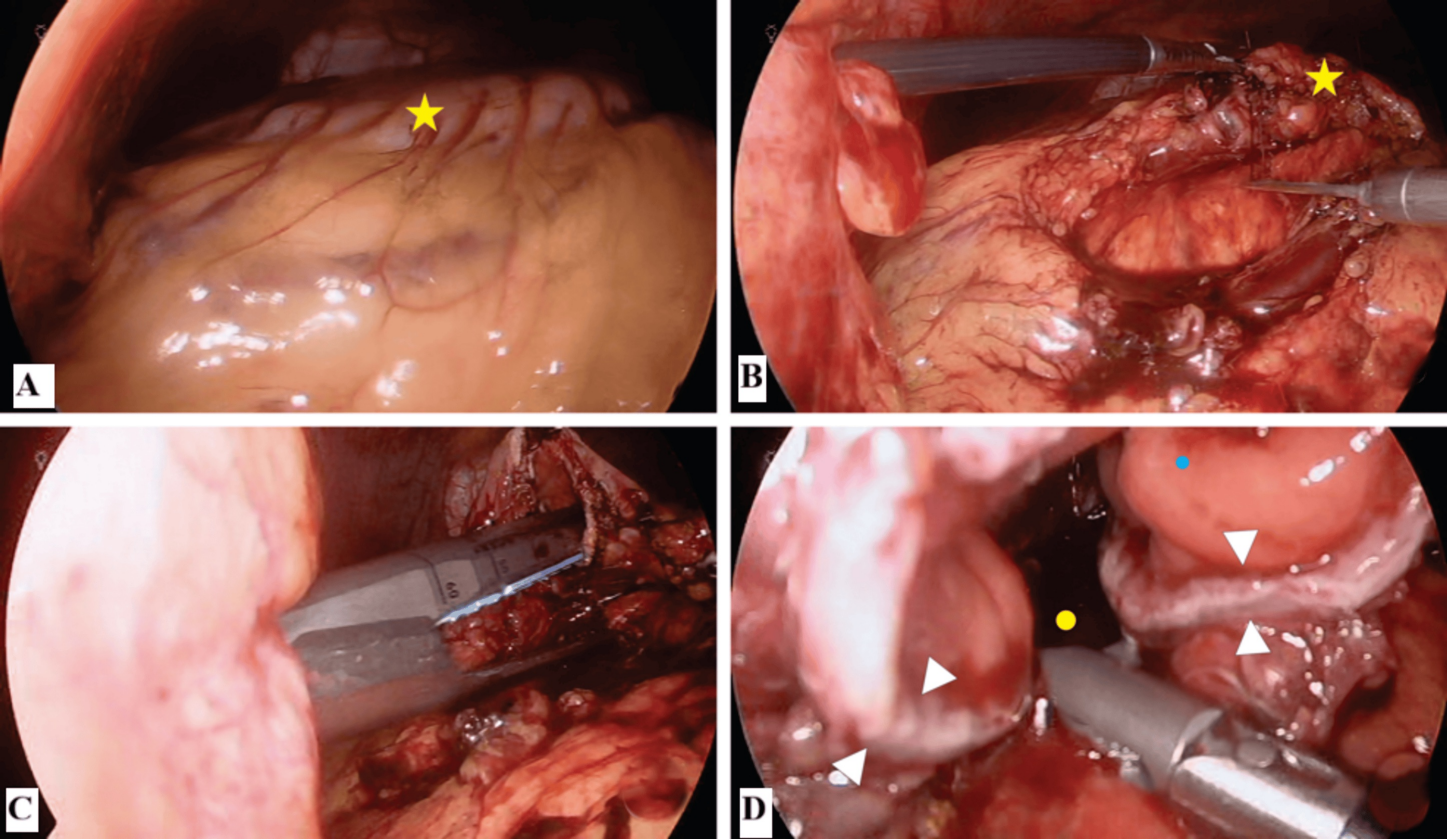
Intraoperative images of the laparoscopic extragastric cystogastrostomy (A) Initial laparoscopic view showing the bulging of the posterior gastric wall (yellow star) caused by the underlying pseudocyst. (B) Dissection and mobilization of the greater curvature of the stomach (yellow star) to expose the pseudocyst wall. (C) Creation of the cystogastrostomy using a linear mechanical stapler. (D) View of the completed anastomosis, showing the communication between the cystic lumen (yellow dot) and the gastric lumen (cyan dot). The white arrowheads indicate the stapled line of the anastomosis

On July 8, 2024, a laparoscopic extragastric cystogastrostomy was performed. A 10 mm right paramedial port, a 5 mm left paramedial port, and a 5 mm left accessory port were placed. A large PFC was identified. The cyst was punctured, draining 600 mL of necrotic material, the presence of which was more consistent with a diagnosis of WON than a simple pseudocyst. A cystogastrostomy was created using an Echelon Flex laparoscopic linear stapler (Figure 2). A 19-Fr Blake drain was placed. Operative time and estimated blood loss were not recorded in the operative note; the anastomosis size was determined by the length of the stapler cartridge. The drain was removed before discharge.

The pathology report described fat necrosis and scant coccoid bacteria. Intraoperative fluid cultures were sent, but the results were not available in the provided records. The patient was discharged but readmitted 20 days postoperatively with a peripancreatic abscess. He was hospitalized and started on a course of broad-spectrum intravenous antibiotics (specific class and duration not specified in the records), with a plan for endoscopic evaluation if there was no clinical improvement. The patient's outcome beyond this readmission is not available.

## Discussion

The management of PPCs must be individualized. The initial treatment for uncomplicated PPCs is conservative, allowing for spontaneous resolution and wall maturation [4,8]. In this case, a six-week waiting period was appropriately observed [2,5]. However, the persistence of symptoms and the giant size of the pseudocyst were clear indications for definitive intervention [3,6].

The choice between EUS-guided and laparoscopic drainage depends on anatomical feasibility, institutional expertise, and cyst characteristics. In our setting, the unavailability of EUS-guided drainage made laparoscopic surgery the primary minimally invasive option. For this patient, a laparoscopic approach was justified by the clinical context: the giant size and the presence of necrotic debris, where a large surgical anastomosis is advantageous [6,9]. Surgical success can be defined by technical success (successful creation of the anastomosis) and clinical success (resolution of symptoms and the collection on imaging). The success rates of surgical drainage are consistently high, ranging from 80% to 97% [5,6,10].

The procedure in our patient was technically successful, achieving complete evacuation of the cavity and the creation of a wide, patent anastomosis. However, clinical success was complicated by the development of a postoperative peripancreatic abscess. Infection is a feared complication, with morbidity rates up to 30% reported in surgical series [5,6]. This outcome does not necessarily reflect a technical failure but rather the severity of the underlying necrotizing process. Our management of this complication, initiating broad-spectrum antibiotics with a plan for potential step-up intervention, follows current recommendations for infected collections [2]. An endoscopic approach through the existing cystogastrostomy could be a viable next step if drainage is required.

## Conclusions

This case illustrates that laparoscopic cystogastrostomy is an effective treatment for giant, symptomatic PFCs, particularly in settings where endoscopic drainage is not available. However, it also highlights the diagnostic challenge of differentiating a PPC from WON preoperatively. Acknowledging the single-case limitation and the possibility that this lesion represented WON, the procedure allowed for successful internal drainage. The postoperative course underscores that even after a technically successful minimally invasive surgery, patients require structured surveillance to promptly manage the infectious complications inherent to severe pancreatitis.
